# Metabolic engineering strategies for the improvement of cellulase production by Hypocrea jecorina

**DOI:** 10.1186/1754-6834-2-19

**Published:** 2009-09-01

**Authors:** Christian P Kubicek, Marianna Mikus, André Schuster, Monika Schmoll, Bernhard Seiboth

**Affiliations:** 1 Research Area Gene Technology and Applied Biochemistry, Institute of Chemical Engineering, TU Vienna, Getreidemarkt, A-1060 Vienna, Austria

## Abstract

*Hypocrea jecorina (= Trichoderma reesei) *is the main industrial source of cellulases and hemicellulases used to depolymerise plant biomass to simple sugars that are converted to chemical intermediates and biofuels, such as ethanol. Cellulases are formed adaptively, and several positive (XYR1, ACE2, HAP2/3/5) and negative (ACE1, CRE1) components involved in this regulation are now known. In addition, its complete genome sequence has been recently published, thus making the organism susceptible to targeted improvement by metabolic engineering. In this review, we summarise current knowledge about how cellulase biosynthesis is regulated, and outline recent approaches and suitable strategies for facilitating the targeted improvement of cellulase production by genetic engineering.

## Background

The β-(1,4)-linked glucose polymer cellulose is a product of the utilisation of solar energy and carbon dioxide by plants and exhibits an annual production of about 7.2 × 0^10^tons. In plants, it is always associated with hemicelluloses, which consist of polysaccharides made up from non-glucose sugars, in which β-1,4-linked xylans and β-mannans make up the major portions, and which also account for a production of 6 × 10^10^tons annually. The degradation of these two polysaccharides is, therefore, a key transformation step in the biological carbon cycle in nature. The ascomycete *Hypocrea jecorina *(anamorph *Trichoderma reesei*) is a saprobic fungus capable of efficiently degrading plant cell wall polysaccharides such as cellulose or hemicelluloses. After identifying it as the cause of a massive infection of cotton-based army material, it was stored at the Quartermaster (QM) collection of the US army at Natick, where its cellulolytic potential was realised in the late 1960s [1]. Several mutant lines were thus derived from the original isolate QM6a and, because of their potent secretion system and high expression level for cellulases and hemicellulases, are used today for the industrial production of low cost enzymes for applications in the pulp and paper, food and textile industries and in the conversion of plant biomass materials into industrially useful products such as sugars and bio-ethanol [2-4].

Cellulases are classified into two broad categories: cellobiohydrolases, whose major activity involves the cleavage of cellobiose residues consecutively from the ends of the cellulose chains, and endoglucanases, whose major activity involves the cleavage of β-glycosidic bonds in the cellulose chain. The members of this system act synergistically and are necessary for the efficient hydrolysis of cellulose to soluble oligosaccharides. However, this classification does not take into account the protein structure and catalytic mechanism, and therefore the 'classification system of carbohydrate active enzymes (CAZy)', developed by Coutinho and Henrissat [5], is today generally accepted and used. Table 1 shows the correspondence of 'old' and 'CAZy' designations for current identified components of the *H. jecorina *cellulase system.

**Table 1:** Nomenclature of cellulolytic enzymes.

**Function**	**Gene**	**Protein**	GH family*
Cellobiohydrolases	*cbh1/cel7a*	CBH1/CEL7A	GH7

	*cbh1/cel6*	CBH2/CEL6	GH6

			

Endo-β-1,4-glucanases	*egl1/cel7b*	EG1/CEL7B	GH7

	*egl2/cel5a*	EG2/CEL5A	GH5

	*egl3/cel12a*	EG3/CEL12A	GH12

	*egl4/cel61a*	EG4/CEL61A	GH61

	*egl5/cel45a*	EG45/CEL45A	GH45

	*cel74a*	CEL74A	GH74

	*cel61b*	CEL61B	GH61

	*cel5b*	CEL5B	GH5

			

β-glucosidases	*bgl1/cel3a*	BGL1/CEL3A	GH3

	*bgl2/cel1a*	BGL2/CEL1A	GH1

	*cel3b*	CEL3B	GH3

	*cel3c*	CEL3C	GH3

	*cel1b*	CEL1B	GH1

	*cel3d*	CEL3D	GH3

	*cel3e*	CEL3E	GH3

*GH family defined according to Coutinho and Henrissat [5].

Recent demands for the production of biofuels from lignocellulose led to a renaissance in *Trichoderma *cellulase research [6-8]. One of the key issues is the generation of improved producer strains. This has traditionally been reached by classical mutagenesis and selection procedures. However, the availability of sophisticated gene manipulation methods and recent elucidation of the genome sequence of *H. jecorina *[9] raised the possibility of introducing molecular genetic methods into such improvement programmes, for example, by manipulating inducer forming pathways, signalling cascades and/or activation of transcription of the cellulase genes. Towards this end, however, a solid understanding of the biochemical basis of cellulase over-production is essential. Interestingly, the genome sequence of *H. jecorina *(*vide supra*) revealed that its set of plant cell wall degrading enzymes is considerably smaller than that of other filamentous fungi. Despite this limitation it nevertheless successfully competes with these other fungi in the hydrolysis of plant material and has gained significant industrial importance. The inability to rationalise this discrepancy underscores our limited understanding of the regulatory principles which govern the synthesis and excretion of *H. jecorina*'*s *cellulases.

In this review, we summarise the current state of knowledge about the mechanisms involved in this regulation, and describe attempts to apply this information to the improvement of cellulase production. The term 'metabolic engineering' will not only be applied to the manipulation of metabolic pathways, but we will also adapt this term for those strategies where the action of transcription factors and signalling proteins has been modified.

### Regulation of cellulase biosynthesis by the natural substrate cellulose

As the prime function of cellulases is the hydrolysis of cellulose thus to provide the fungal cells with soluble oligomers for further catabolism, these enzymes should be formed in the presence of cellulose. While this is indeed the case, most *H. jecorina *cellulases are adaptive enzymes, that is, their transcripts are not formed during growth on monosaccharides and their full expression requires the presence of an inducer. Since the natural inducer cellulose is insoluble, several studies were performed to determine how an insoluble polymer, which cannot traverse the cell membrane, would initiate cellulase production. Several lines of explanation were offered, most of which emphasised the formation of a low molecular weight and soluble inducer from cellulose: one of them [10,11] postulates that a low basal level of cellulases (mainly CEL7A and CEL6A) is formed, which can start the degradation of cellulose and thereby release small amounts of oligosaccharides, which can induce further cellulase biosynthesis. Evidence for this theory was provided by antibody competition and antisense RNA experiments [10,11]. More recently, Foreman et al. [12] identified several further genes that displayed regulatory patterns consistent with the possibility that they play a role in primary inducer formation for cellulase expression. Among them, the mRNA of *cel5b *was moderately expressed during growth on glycerol, glucose, sophorose and lactose, and only slightly induced over this level by cellulose. Also CEL5B contains the consensus sequence for membrane-anchoring via a glycosylphosphatidylinositol residue. All these properties make it an interesting candidate for generating the inducer of cellulase formation.

Another hypothesis also supported by experimental evidence, emphasises the fact that conidia of *H. jecorina *contain surface-bound cellulolytic activity [13,14]. Removal of this activity by non-ionic detergents impairs germination of the conidia on cellulose. Interestingly, CEL6A is the predominant cellulase on the conidia, and they completely lack the endoglucanase CEL7B [14]. The possibility of improving cellulase production by engineering the conidial CEL6A amount was suggested by the findings that the hyper-producer strain *H. jecorina *RUT C-30 exhibited an elevated conidial level of CEL6A. Introduction of multiple copies of the *cel6a *gene into *H. jecorina *in fact led to an enhanced secretion of both CEL7A and CEL6A on cellulose, and the transformants showing highest cellulase activity on cellulose also appeared to contain the highest level of conidial-bound CEL6A [14,15]. Consistent with these data, a *cel6a *knockout strain exhibited a pronounced lag in growth on cellulose and cellulase formation [16]. The major role of *cel6a *in the primary attack on cellulose was further substantiated by Seiboth et al. [17], who compared isogenic strains in which the corresponding genes of the main cellulases (*cel6a, cel7a, cel7b, cel5a*) had been deleted. Strains in which *cel6 *and *cel5a*, respectively, had been deleted showed a significantly reduced expression of the remaining cellulase genes, whereas in strains carrying the *cel7a *or *cel7b *deletion, these transcripts were clearly detectable. A strain in which both the cellobiohydrolases *cel6a *and *cel7a *had been deleted, however, was unable to initiate growth on cellulose. During growth on lactose (a soluble carbon source also provoking cellulase gene expression, see below), these strains showed no significant alterations in their ability to express the respective other cellulase genes. Taken together, these data provide significant support for the role of CEL6A and other conidial-bound cellulases (such as CEL5A, for which a conidial location is not yet known) in the induction of cellulases and germination on cellulose when a conidial inoculum is used.

A third line of explanation extends the findings that cellulase transcripts have been detected in cultures of *H. jecorina *grown on glucose for 20 to 30 hours after its consumption [17]. This phenomenon is unrelated to relief from carbon catabolite repression (see below), and it is also not due to starvation because simple incubation in media lacking any carbon source does not lead to cellulase transcription. Thus these findings are still lacking a consensus interpretation, but it is possible that an inducing sugar is derived from carbohydrates released from the fungal cell under starvation conditions.

While these three explanatory models are in essence not really different and rather may reflect mechanisms operating under different physiological conditions, they all imply that the action of these cellulases leads to the formation of an inducer of cellulases. In fact, the most strongly inducing component, the β-1,2-diglucoside sophorose, was originally isolated from culture fluids of *H. jecorina *[18] and has been shown to be formed during growth of *H. jecorina *on cellobiose [19], and after hydrolysis of cellulose with the *H. jecorina *cellulase system by transglycosylation [20]. Induction by sophorose is complex and affected by various parameters such as its concentration and rate of uptake [21,22]. Kubicek et al. [23] showed that sophorose is taken up by a cellobiose permease, which also transports several cellooligosaccharides. The permease has a low K_m _but also only a low V_max _for sophorose, and thus competes with the extracellular β-glucosidase, which has a much higher K_m _but also V_max _for it. This implies that sophorose transport is favoured at low concentrations. Loewenberg and Chapman [24] arrived at similar conclusions from a study of the interrelationship between catabolism of sophorose and cellulase induction. They claimed two pathways of sophorose utilisation: a catabolic pathway that has a high capacity but low affinity for sophorose; and a cellulase inducing pathway having a lower capacity but higher affinity for sophorose. Unfortunately, the permease gene has not been identified so far.

As for the enzyme forming sophorose, most authors implied a β-glucosidase in this process. *H. jecorina *has been reported to produce extracellular [25], cell wall-bound [26], plasma membrane-bound [27] and intracellular [28,29] β-glucosidases. Experimental evidence suggests that the extracellular and a major part of the cell wall-bound activities are due to the same enzyme [26]. The gene *cel3a *[25,30], encoding the major extracellular β-glucosidase, has been investigated in this direction: the disruption of the *cel3a *gene resulted in a delay in induction of the other cellulase genes by cellulose, but not by sophorose. A *cel3a*-multicopy strain formed higher amounts of cellulases than the parent strain under non-saturating concentrations of sophorose, but both strains were comparably efficient at saturating concentrations [31]. However, the β-glucosidase inhibitor nojirimycin strongly inhibited cellulase induction in all strains including the *cel3a *disrupted strain, suggesting that the CEL3A is not the only β-glucosidase involved in inducer formation [31]. Saloheimo et al. [29] described an intracellular β-glucosidase, CEL1A, producing mainly cellotriose from cellobiose and sophorose and cellobiose from glucose, which could therefore be involved in inducer formation. However, no data on the engineering of its expression and the effect on cellulase gene expression have yet been reported.

### Transcriptional regulation of cellulase gene expression

The obligatory presence of an inducer for cellulase gene expression to occur implies tight regulation of the respective promoters. In fact, most of the cellulase genes are regulated in a consistent way, although the relative ratio of their expression is somewhat different in higher producer mutants [12], probably due to promoter titration effects. The identification of genes involved in transcriptional regulation of cellulase gene expression has been a major effort in the past 10 years. Today, three positive transcriptional activators (XYR1, ACE2 and the HAP2/3/5 complex) as well as two repressors (ACE1 and the carbon catabolite repressor CRE1, see below) have been demonstrated to be involved in this regulation.

XYR1 (xylanase regulator 1), a zinc binuclear cluster protein binding to a GGCTAA-motif arranged as an inverted repeat, is the general main activator of cellulase and hemicellulase gene expression [31]. It is an orthologue of the *xlnR *gene of *Aspergillus niger *[32]. Consensus sequences for XYR1 have been found in all inducible *H. jecorina *cellulase promoters (unpublished data). Deletion of *xyr1 *eliminates cellulase induction on cellulose and sophorose, thus proving its essential role in the induction process [31]. *Xyr1 *transcription seems not to be induced during growth on cellulose [33]. Whether an increase in constitutive expression of *xyr1 *would increase enzyme formation is not sufficiently understood. Aigner-Mach et al. [33] fused the *xyr1 *gene under the regulatory signals of the *nag1 *(N-acetyl-β-D-glucosaminidase) promoter, which resulted in a slightly earlier beginning of xylanase formation but did not significantly enhance the final enzyme titre. However, these studies used the uninduced, basal expression level of *nag1*, which is not much higher than that of *xyr1 *itself, and studies using stronger expressed promoters (such as those for glycolytic or hydrophobin genes) must be used to clarify whether the constitutive expression of *xyr1 *would enhance cellulase and/or xylanase formation.

The second characterised cellulase activator ACE2 also encodes for a protein belonging to the class of zinc binuclear cluster proteins found exclusively in fungi [34]. It has so far been shown to occur only in *Trichoderma *spp. During growth on cellulose, deletion of the *ace2 *gene led to lowered induction kinetics of cellulase mRNAs and 30% to 70% reduced cellulase activity [34,35]. Interestingly, cellulase induction by sophorose was not affected by *ace2 *deletion [34]. The DNA-binding domain of ACE2 was shown to bind *in vitro *to the 5'-GGCTAATAA site present in the *cbh1 *promoter. Therefore, both XYR1 and ACE2 are able to bind the complete motif. Stricker et al. [35] suggested that phosphorylation as well as dimerisation are prerequisites for binding ACE2 to its target promoter.

In addition, expression from the *cel6a *promoter has been shown by promoter mutation and *in vivo *footprinting analysis to be dependent on a CCAAT box bound by the HAP2/3/5 protein complex [36]. Zeilinger et al. [37] cloned the corresponding *hap2, hap3 *and *hap5 *genes from *H. jecorina*, and showed that they encode proteins whose core regions display great similarity to Hap homologues from other organisms. All three of the *H. jecorina *HAP proteins were essential for binding to the CAE (*cbh2*-activating element) in the *H. jecorina cel6a *promoter [37]. The CCAAT motif is found in approximately 30% of the 5'-non-coding regions of eukaryotic genes [38]. In analogy to the mammalian NF-Y complex containing NF-YA, NF-YB and NF-YC orthologues of HAP2, HAP3 and HAP5, respectively, they contain a histone fold motif, a structural feature of histones suggesting that NF-Y might be involved in the organisation of the chromatin structure [39]. Thereby the action of acetyltransferases may play a role in the local disruption of nucleosomes since an association of GATA-1 and NF-Y with acetyltransferases p300/CBP has been shown [40,41].

The hypothesis that the CCAAT sequences in the cellulase promoters could play a conserved role in the generation of an open chromatin structure necessary for full transcriptional activation is supported by the detection of a nucleosome-free region around the XYR1/ACE2/HAP2/3/5-binding area in the *cel6a *promoter, which is flanked by strictly positioned nucleosomes [42]. Induction by sophorose results in a loss of positioning of nucleosomes -1 and -2 downstream of the binding area, thus making the TATA box accessible. A mutation in the CCAAT box shifted this positioning, thus proving the role of the HAP2/3/5 complex in this process [42].

ACE1 contains three Cys_2_His_2_-type zinc fingers and was shown to bind *in vitro *to eight sites containing the core sequence 5'-AGGCA scattered along the 1.15-kb *cel7a *promoter [43]. Deletion of *ace1 *resulted in an increase in the expression of all the main cellulase and hemicellulase genes in sophorose- and cellulose-induced cultures, indicating that ACEI acts as a repressor of cellulase and xylanase expression [44] and of *xyr1 *during growth on D-xylose [33]. A strain bearing a deletion of both the *ace1 *gene and *ace2 *gene expressed cellulases and xylanases similar to the Δ*ace1 *strain, probably due to the remaining activity of XYR1 [44].

Interestingly, ACE1 has been identified as an orthologue of the *Aspergillus nidulans stzA *gene encoding a stress response factor [45]. The authors provided evidence of competition, or interaction, between the ACE1/StzA and AreA binding sites in promoters of *stzA *and its orthologues, and in genes involved in the metabolism of amino acids. The *A. nidulans *and *A. fumigatus cpcA *(cross pathway control regulator of amino acid biosynthesis) promoters have seven potential ACE1/StzA binding sites, six of which are highly conserved in position. The presence of potential CPC1 binding sites (5'-TGAC/GTCA) in the *stzA *and *ace1 *promoters suggests an intriguing link between intracellular amino acid availability and cellulase gene expression. In accordance with these findings a recent study by Gremel et al. [46] indeed revealed that cellulase gene expression can be enhanced by the addition of methionine.

Summarising these findings, one can hypothesise that the substrate-unspecific activator XYR1 is fine-tuned by more specific transcriptional regulators such as ACE1 and ACE2. This working model concurs with the findings that XYR1 binds to an inverted repeat either as a homo- or a heterodimer, respectively, thereby providing the opportunity for specific regulatory proteins to interact with the accordant promoter and/or XYR1. The role of the HAP2/3/5 complex in this regulation may be that of a general transcriptional enhancer raising the accessibility of the other factors to the cellulase promoters.

### Carbon catabolite repression

As mentioned above, expression of a large majority of the cellulase genes that have been studied in *H. jecorina *and other filamentous fungi does not occur during growth on glucose. This has been shown to be due to both inducer exclusion (that is, inhibition of inducer [= sophorose] uptake by D-glucose [23]) and glucose repression [17,42,47]. The latter specifies a transcriptional regulation controlling the preferential use of substrates such as D-glucose or other monosaccharides whose catabolism provides a high yield of ATP and is more generally called carbon catabolite repression (CCR).

Consequently, one of the earliest attempts for engineering cellulase production was removal of carbon catabolite repression. Classical mutagenesis combined with selection for 2-desoxyglucose resistance (an agent believed primarily to enrich carbon catabolite-resistant mutants [48]) has led to increased cellulase producers such as *H. jecorina *RUT C30 [49], RL-P37 [50] and CL847 [51]), thus supporting the possible importance of CCR in cellulase formation. However, later molecular genetic analyses showed that *T. reesei *is generally less affected by CCR than, for example, *Aspergillus *and that the cellulase and xylanase genes are mainly affected at their low constitutive level, their induction being only partially affected [42], and consequently these mutants did not form much cellulase on D-glucose (see below).

In *Trichoderma/Hypocrea *and other ascomycetous fungi, the key player in this glucose repression is the Cys_2_His_2 _type transcription factor CREI/CreA/[52,53]. Interestingly, *H. jecorina *RUT C30 was indeed shown to contain a truncation in the *cre1 *gene [52]. It is located on scaffold 2: 786955-789433 (ID 120117), and the mutant is characterised by a loss of a 2478-base pair fragment, which starts downstream of the region encoding the CRE1 zinc finger and reaches into the 3'-non-coding region [54]. However, because CCR of cellulase gene expression is only partial (*vide supra*), cultivation of this mutant on D-glucose results in only low cellulase levels, and hyper-production is still inducer dependent.

The binding consensus motif for *A. nidulans *CreA was determined to be 5'-SYGGRG [55]. *In vivo *functionality of the CRE1 binding sites have been shown for the *cbh1 *and *xyn1 *promoters of *H. jecorina *where mutations in the binding sequences led to constitutive expression of these genes in the presence of D-glucose [47,56]. Functional CREI/CreA binding sites have been shown to consist of two closely spaced 5'-SYGGRG motifs, and it has been suggested that direct CREI/CreA repression would occur only through such double binding sites. Phosphorylation of a serine in a conserved short stretch within an acidic region of *H. jecorina *CREI has been demonstrated to regulate its DNA binding [57]. Phosphorylation of this serine may involve a casein kinase 2. Casein kinases of this class are known from various other organisms to play a role in the regulation of a large number of transcription factors [58]. The SNF1 kinase, which plays a central role in the regulation of CCR in yeasts [59], appears not to be involved in the phosphorylation of CRE1 in *H. jecorina *[60].

In addition to *creA*, *A. nidulans *is known to contain three further genes, *creB, creC *and *creD*, which participate in CCR [61-64]. Respective orthologues are also present in the *H. jecorina *genome (Table 2). CreB encodes a deubiquitinating enzyme and is a functional member of a novel subfamily of the *ubp *family defined by the human homologue UBH1 [62]. It forms a complex with a WD40-repeat protein encoded by *creC *[63], which is required to prevent the proteolysis of CreB in the absence of CCR [61]. Disruption of the *creB *homologue *cre2 *in *H. jecorina *led to deregulation of genes normally subject to CCR [64]. Interestingly, the E3 ubiquitin ligase LIM1 also responds to cellulase inducing conditions and binds to the *cbh2*-promotor [46].

**Table 2:** *Hypocrea jecorina *orthologues of *creB, creC *and *creD*.

***Aspergillus nidulans *gene**	***Hypocrea jecorina *orthologue**	**Genomic location**	**Protein ID**	**% Identity**	**Score**	**Negative probability**
*creB*	*cre2*	scaffold_12:339018-341830	122405	45	1455	e-120

*creC*	*cre3*	scaffold_14:854865-857183	64608	48	1424	e-118

*creD*	*cre4*	scaffold_26:422136-424237	81690	39	915	e-84

Mutations in *creD *suppress the phenotypic effects of mutations in *creC *and *creB *[65]. CreD contains arrestin domains and PY motifs and is highly similar to *Saccharomyces cerevisiae *Rod1p and Rog3p, which interact with the ubiquitin ligase Rsp5p [66]. Deubiquitinating enzymes are cysteine proteases, and the most common role for ubiquitin is to target proteins for degradation by the proteasome. Recently, the activation domains of certain transcription factors have been demonstrated to serve as direct targets for ubiquitylation, and it has been hypothesised that modulation of activation domains by the ubiquitylation level provides an important mechanism for the regulation of gene transcription [67]. It is tempting to speculate that this explanation may extend to catabolite repression in *H. jecorina*.

The way in which the presence of glucose triggers CCR is still only poorly understood in filamentous fungi. In *S. cerevisiae*, the D-glucose and D-fructose phosphorylating enzymes are also involved in D-glucose and carbon catabolite sensing: it has three hexose-6-phosphorylating enzymes including two hexokinases and one glucokinase. Each of them enables *S. cerevisiae *to grow on D-glucose, but the hexokinase Hxk2p is responsible for the main enzymatic activity and glucose repression mediated by the carbon catabolite repressor Mig1p (whose DNA-binding domain is highly similar to that of CRE1) [68-70]. The mechanism by which Hxk2p contributes to glucose repression has not yet been fully elucidated, but its catalytic activity seems to be dispensable and thus signal transmission may rather depend on substrate binding-induced conformational changes in the Hxk2p protein or a direct regulatory role of the Hxk2p in the nucleus (discussed, for example, in Linhoff et al. [39]). In *A. nidulans *only a single glucokinase and a single hexokinase are present. Flipphi et al. [71] showed that only mutations in both kinase genes lead to CreA-mediated carbon catabolite derepression. Similarly, *H. jecorina *features one glucokinase (GLK1) and one hexokinase (HXK1), and CCR by D-glucose and D-fructose is retained in both single deletion strains whereas Δ*glk1/*Δ*hxk1 *strains are derepressed (L Hartl, CP Kubicek and B Seiboth, Carbon signaling by hexose phosphorylating enzymes in *Hypocrea jecorina, submitted***)**. The level of derepression in Δ*glk1*Δ*hxk1 *strains was higher compared with the Δ*cre1 *mutant RUT C30, thus contrasting findings in *A. nidulans *[71].

### Engineering lactose utilisation

In addition to cellulose-containing plant polysaccharide mixtures, D-galactosyl-β-1,4-D-glucoside lactose is able to induce cellulase gene expression. The obvious advantage of lactose over cellulose is that it is soluble and, therefore, provides a preferred carbon source for the production of recombinant proteins driven by cellulase (for example, *cel7a*) promoters. However, lactose catabolism is slow and cellulase yields produced on lactose are somewhat lower than those on cellulose [72], thus making strain improvement by metabolic engineering even more attractive.

One of the enigmas of cellulase production on lactose is the fact that lactose occurs naturally only in the milk of mammals and accumulates in large quantities only as a by-product of cheese production in whey. Consequently lactose is unlikely to be a carbon source normally found in the habitat of a fungus like *Trichoderma *spp with a saprobic and mycoparasitic life style. Probably, the β-galactosidases involved in the initial hydrolysis of lactose into D-galactose and D-glucose have other roles in fungal metabolism, such as the cleavage of D-galactose residues from glycosylated proteins or from polymeric structures found in different plant or fungal cell walls. Substrate patterns and kinetics of the extracellular GH family 35 β-galactosidase BGA1 of *H. jecorina *support the latter role [73].

Lactose catabolism in *H. jecorina *is initiated by the extracellular hydrolysis of lactose in its monomers D-galactose and D-glucose, mainly by the BGA1 β-galactosidase and also by a second, not yet identified and mainly cell wall-bound, β-galactosidase [74]. This is in contrast to the situation in *A. nidulans *and *Kluyveromyces lactis *where uptake of lactose occurs by a specific lactose permease followed by subsequent intracellular hydrolysis [75]. Both the lactose permease and the intracellular GH family 2 β-galactosidase are absent from the genome of *H. jecorina *[76]. The rate of the extracellular lactose hydrolysis seems to be critical for cellulase gene expression: whereas a lowering of its rate (by deletion of the major extracellular BGA1) affected growth but not cellulase induction, constitutive overexpression of *bga1 *lead to high growth rates on lactose but completely abolished cellulase induction [76].

As a result of the extracellular hydrolysis, D-galactose and D-glucose are taken up and are then channelled in the different pathways for their degradation. The extracellular hydrolysis leads to an interesting question: if cellulases are induced by lactose and lactose is cleaved extracellularly, then are their monomers also able to induce cellulase induction? The answer is no. During normal growth, neither D-glucose nor D-galactose nor mixtures of them are able to induce cellulase transcription, and the same effect was obtained in the *cre1*-negative background [77]. Some induction by D-galactose occurred at low growth rates in a carbon-limited chemostat during growth on D-galactose and a mixture of D-galactose and D-glucose, whereas no induction was apparent under the same conditions with D-glucose as a limiting carbon source [78]. Nevertheless, cellulase expression was significantly lower than during growth on lactose at the same growth rate, thus implying that lactose is still a superior inducer than D-galactose or a mixture of D-galactose and D-glucose.

So what could be the difference between D-glucose and D-galactose arising from the action of β-galactosidase and a mixture of D-galactose and D-glucose? The key to understanding this phenomenon may be found in the stereospecificity of the D-galactopyranose cleaved by BGA1 from lactose: in contrast to the D-glucose moiety, which can be either in the α- or β-form, D-galactose arising by BGA1 hydrolysis is in the β-anomeric form. This β-anomer will be converted to the α-anomer by chemical mutarotation, but this is a slow process. Therefore, many organisms have developed aldose 1-epimerases (mutarotases), which enzymatically enhance the rate of chemical mutarotation. The biological importance for this enzymatic mutarotation lies in the fact that the Leloir pathway is the main or exclusive pathway for the assimilation of D-galactose by most microorganisms [79]. This pathway involves the subsequent operation of galactokinase (GAL1; EC 2.7.1.6), D-galactose-1-phosphate uridylyl transferase (GAL7; EC 2.7.7.12) and UDP-galactose 4-epimerase (GAL10; EC 5.1.3.2) to convert D-galactose to D-glucose 1-phosphate, which by the action of phosphoglucomutase (EC 2.7.5.1) is transformed to D-glucose-6-phosphate, an intermediate of glycolysis [78]. The galactokinase, however, only phosphorylates the C1 of the α-D-galactose [80]. Proof for the importance of this reaction comes from deletion of the mutarotase encoding gene in *E. coli*, which consequently resulted in a significantly decreased growth rate on lactose [81].

The genome of *H. jecorina *contains three putative aldose 1-epimerase genes (*aep1-3*), of which two encode intracellular proteins (AEP1-2) and one a putative extracellular protein (AEP3). However, none of these genes was expressed during normal growth on lactose and consequently no mutarotase activity could be detected during growth on lactose [82]. This implies that enzymatically catalysed mutarotation of β-D-galactose is either absent or inefficient and the operation of the Leloir pathway thus depends mainly on the availability of α-D-galactose from chemical mutarotation.

To prove that the availability of β-D-galactose arising from lactose may thus be the relevant parameter in the induction of cellulase gene expression, the C-terminal aldose 1-epimerase domain of the *S. cerevisiae *Gal10p was introduced into *H. jecorina *and its effect on lactose metabolism and cellulase gene expression studied [82]. This manipulation resulted in increased growth rates on lactose and to significant down-regulation of cellulase gene transcription. Both findings were copy-number dependent. Consequently, β-D-galactose appears to be an important intermediate in the induction of cellulases by lactose.

**Figure 1:**
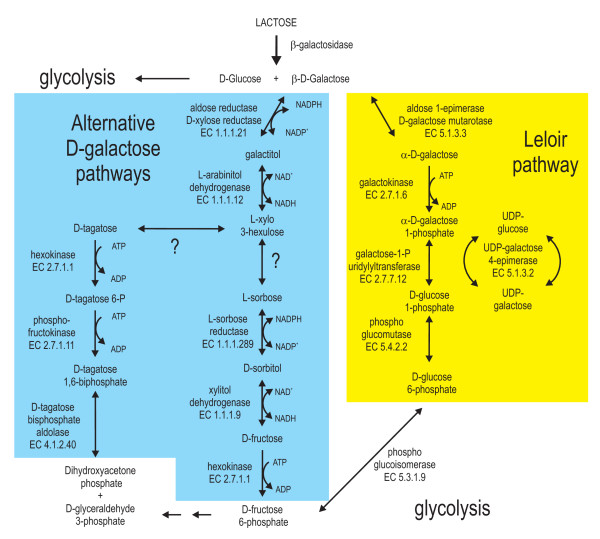
**Lactose and D-galactose catabolism in *Hypocrea jecorina***. The heterodisaccharide lactose is cleaved extracellularly into its monomers D-glucose and D-galactose. While D-glucose is assimilated via glycolysis (not shown), D-galactose can be converted by two different pathways. The galactokinase of the classical Leloir pathway (left) is specific for α-D-galactose and therefore, β-D-galactose has to be epimerised to the α-anomer before it can enter this pathway. A second pathway identified in *Hypocrea jecorina *starts with the reduction of both anomeric forms of D-galactose to galactitol. Two hypothetical pathways are drafted for the further degradation of galactitol.

The biochemical pathway by which *H. jecorina *then metabolises the β-anomer of D-galactose has been the subject of intensive investigations in past years: it starts by a reduction of D-galactose to galactitol by the nicotinamide adenine dinucleotide phosphate-dependent D-xylose reductase (EC 1.1.1.21) XYL1 [74]. Knockout experiments proved that XYL1 is the main aldose reductase activity for D-galactose catabolism, and also for catabolism of the pentoses D-xylose and L-arabinose [83]. Also, two other enzymes involved in the D-xylose/L-arabinose catabolic pathway participate in this alternate pathway of D-galactose utilisation, that is, L-arabinitol dehydrogenase LAD1 (EC 1.1.1.12) and xylitol dehydrogenase XDH1 (EC 1.1.1.9). However, the product of both enzymes *in vitro *is D-xylo-3-hexulose [[84,85]; unpublished data], whose further catabolism is still unknown. In *A. nidulans *L-sorbose was shown to be an intermediate of the second D-galactose pathway. If and how D-xylo-3-hexulose is converted to L-sorbose is not known. Further catabolism of L-sorbose is known, however, it is reduced to D-sorbitol, followed by oxidation to D-fructose and finally phosphorylated to D-fructose-6-phosphate. The latter is likely catalysed by the hexokinase HXK1, because a knockout in the corresponding *hxk1 *leads to an inability to grow on galactitol (L Hartl and B Seiboth, unpublished data). L-Sorbose reduction to D-sorbitol can be catalysed by an L-sorbose reductase. The L-xylulose reductase LXR1 is able to perform the *in vitro *reaction [86], however, a knockout in *lxr1 *has no effect on galactitol or lactose utilisation (B Metz, R de Vries, S Polak, V Seidl, B Seiboth, The *Hypocrea jecorina *(syn. *Trichoderma reesei*) *lxr1 *gene encodes a D-mannitol dehydrogenase and is not involved in L-arabinose catabolism, *submitted*), and therefore the reductase actually involved still has to identified. The oxidation of D-sorbitol to D-fructose can be catalysed by both LAD1 or XDH1 [84,85]. Still other pathways of D-galactose assimilation cannot be excluded (Figure 1).

The relative importance of this alternate catabolic route seems to differ between fungi since in *A. nidulans *the pathway can fully compensate for the loss of the Leloir pathway [87], while in *H. jecorina *inactivation of the Leloir pathway leads to strains which are significantly impaired in their growth on D-galactose [79]. One major contribution of this pathway for lactose catabolism is the generation of the BGA1 inducer galactitol [88].

The putative involvement of L-sorbose as an intermediate of the alternate pathway is intriguing as L-sorbose has been found to regulate co-ordinately the cellulase genes at transcriptional level [89]. This makes the identification of the genes acting downstream in the alternate pathway an important point for potential strain improvement.

### Regulation of lactose induction of cellulase gene expression

Evidence described above points towards a major role of the β-D-galactose anomer in cellulase induction by lactose. Yet the actual mechanism must be more complex: despite the fact that D-galactose generated from lactose must be catabolised via the alternate pathway, disruption of the *gal1 *gene results in a strong decrease of cellulase formation on lactose [77]. In these Δ*gal1 *strains, cellulase induction can be restored by retransformation with the structurally unrelated galactokinase gene from *E. coli *but cannot be restored by the introduction of an enzymatically inactive galactokinase [90]. Therefore, galactokinase activity itself is important for cellulase induction by lactose. Interestingly, inactivation of the subsequent step, in which GAL7 transforms D-galactose-1-phosphate to UDP-galactose, has no effect on cellulase induction except that the cellulase transcripts have a longer half-life [91]. Together, these data would suggest that the concentration of D-galactose-1-phosphate is important for cellulase induction. However, *H. jecorina *(as well as other fungi) also contains an UDP-galactose pyrophosphorylase (scaffold_1:393507-396492; EC 2.7.7.10), which may compensate for a loss of *gal7*, and whose role in cellulase induction has not yet been clarified.

In addition, a knockout in *xyl1 *(encoding the aldose reductase XYL1) also results in a decrease in cellulase gene expression on lactose, although not as severe as a knockout in *gal1 *[83]. A *xyl1/gal1*-double knockout does not result in an additive effect but remains at the level of the *gal1 *knockout, thus indicating that a blockage in either pathway acts on the same target [83]. A consensus explanation for these findings would be that the inducer formed during lactose catabolism is an oligosaccharide composed of metabolites both from the Leloir and the alternate pathway. To test this hypothesis, we recently performed a metabolomic analysis of intracellular oligosaccharides formed in *H. jecorina *QM 9414 (parent strain) and the various pathway deletion strains (manuscript in preparation). Indeed, several oligosaccharides were detected, whose intracellular concentrations changed in a consistent way with cellulase formation. Further investigations are needed, however, to prove that any of these functions as an inducer of cellulase gene expression by lactose.

XYR1, the regulator of cellulase formation on cellulose, has also been shown to be the major regulator of their induction by lactose [92]. In addition, x*yr1 *deletion strains are almost completely unable to grow on lactose because *xyr1 *is essential for the induction of both *xyl1 *and *bga1*.

### Modification of signal transduction pathways - an alternative approach to enhance cellulase formation?

Filamentous fungi, such as *H. jecorina *have to deal with countless challenges to succeed in the battle for nutrients, space and reproduction in the rich habitat of a tropical rain forest. To this end, all organisms have developed sensitive tools which enable them to receive extracellular signals and fine-tune their gene expression and metabolism accordingly. Since *Trichoderma *spp. dominantly occupy their ecological niches, it is reasonable to assume to the presence and operation of efficient machinery for the perception and interpretation of environmental signals. Thus a better understanding of signal transduction pathways initiating and/or modulating this process may help to develop new strategies for improving cellulase gene expression.

### Light as a signal influencing cellulase formation

Light is a fundamental abiotic factor which influences most living organisms. As a signal, light is of the utmost importance, reflected in the presence of circadian rhythms, which can be reset by light and enable anticipation of changing conditions according to day and night (for example, in terms of ultraviolet light, temperature or humidity) [93]. These circadian rhythms as well as light impact on the transcription of a considerable number of genes [94,95]. *Neurospora crassa *has become a paradigm for investigation of light response and circadian rhythmicity. Therefore functions, mechanisms and regulatory processes connected with circadian rhythms and light response in this fungus are well documented [96-98]. Two of the most important factors of these processes in *N. crassa *are the two photoreceptors WC-1 (white-collar-1) and WC-2 (white collar 2). WC-1 and WC-2 can interact via their PAS domains and both proteins form the WCC complex. A further important member of this regulatory circuit is the photoreceptor VIVID, a small blue-light photoreceptor, which is induced by the light-activated WCC complex. Output pathways of the respective regulatory circuit analysed so far have been limited to those involved in dealing with the harmful effects of sunlight [95].

There are also several studies available for *Trichoderma *spp., which provide first insights into the regulation of light response. However, output pathways besides those directly related to the effect of light have only recently gained attention. In search of signal transduction pathways involved in cellulase gene expression, a screening for genes differentially expressed in a cellulase non-inducible mutant strain and the parental/reference strain QM9414 revealed several candidate genes to be studied further [99]. Unexpectedly, a gene putatively involved in light response, later named *env1 *(encoding ENVOY for 'messenger'), was among these genes. ENVOY represents the *H. jecorina *which is an orthologue of the blue-light photoreceptor VIVID [100] from *N. crassa *and is the first signal transduction component studied on a molecular level in *H. jecorina*. Support for this claim comes from the findings that a mutant strain lacking the PAS-domain of ENVOY (*env1*^*PAS*-^) shows a severe growth defect in light, but grows normally in darkness, and transcription of *env1 *is clearly induced by light. Nevertheless, ENVOY could not complement a mutant in which VIVID was not functional [100]. Transcription of the cellobiohydrolase gene *cel7a *is significantly enhanced upon cultivation on cellulose in constant light compared with constant darkness in *H. jecorina*. However, despite this function, ENVOY is not solely responsible for integration of the light signal into the regulatory mechanism of cellulase gene expression [100]. Results from shake flask experiments showed enhanced cellulase activity in delta-*env1 *mutants, and these results were confirmed in laboratory scale fermentations (M Gyalai-Korpos and M Schmoll, unpublished). Preliminary experiments with strains deleted in other components of the light signalling pathway, that is, the orthologues of the *N. crassa *photoreceptors WC-1 and WC-2, BRL1 and BRL2, respectively, confirm the effects found with *env1 *(M Schmoll, unpublished results).

In *N. crassa*, the number of regulatory targets (7% of the genome) of the light signalling proteins exceeds those of the genes whose expression actually responds to light (3%) [94], which suggests functions of these proteins beyond responding to light. Consistent with this *H. jecorina *ENVOY appears to exert several additional functions beyond light signalling [101], which warrants a deeper investigation of the role of these proteins in the physiology of *H. jecorina*.

### Heterotrimeric G-protein signalling

The signalling pathway of heterotrimeric G-proteins [102] is involved in diverse cellular functions in fungi, for example, regulation of growth, germination, production of antifungal metabolites, mycoparasitic coiling, conidiation, and sexual and vegetative development [103-108]. The genome of *H. jecorina *comprises three G-alpha subunits, one G-beta subunit and one G-gamma subunit [109]. In the inactive state G-alpha, G-beta and G-gamma subunits form a complex bound to their cognate G-protein coupled receptor (GPCR). Upon reception of an environmental signal, this membrane-bound GPCR transmits the signal to this complex by altering its conformation and releasing the trimeric complex: GDP bound by the G-alpha subunit is exchanged for GTP and the complex divides into two parts. One part is the alpha subunit with the GTP and the second part is the G-beta-gamma heterodimer. Both parts are then free to interact with their specific effectors.

**Figure 2:**
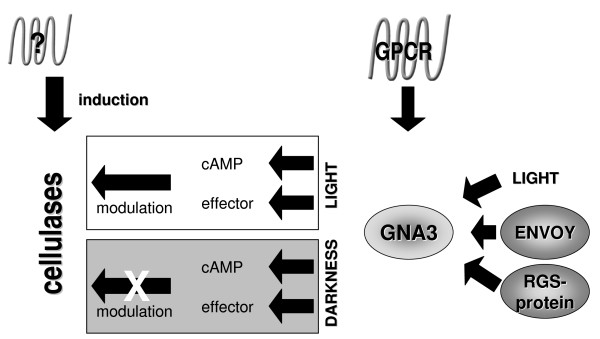
**Schematic model of the proposed function of GNA3**. Upon activation by its cognate G-protein coupled receptor (GPCR), GNA3 causes increased cAMP-levels and acts on its downstream effector. These events result in positive modulation of cellulase gene transcription, the induction of which is initiated by an as yet unidentified pathway. Transcription of *gna3 *is enhanced by light, negatively regulated by ENVOY and activation of GNA3 is decreased by a regulator of the G-protein signalling protein. The GNA3 downstream pathway leading to modulation of cellulase gene transcription is perturbed in darkness.

One of the signals frequently linked with the G-protein pathway is the activation of adenylate cyclase and subsequent activation of protein kinase A by cyclic AMP [110]. In fact earlier studies have reported that cAMP enhances cellulase biosynthesis [110]. Also, in *Cryphonectria parasitica*, a class I G-alpha subunit (CPG-1) has been reported to be necessary for cellulase gene expression [111]. In *H. jecorina*, the potential roles of two G-proteins, GNA1 and GNA3, in cellulase gene expression have been investigated. Studies with strains expressing the constitutively activated G-alpha subunit GNA3m as well as antisense and sense-mutant strains of *gna3*, revealed that this G-protein positively influences cellulase gene expression in constant light, but not in darkness. Accordingly, the light regulatory protein ENVOY negatively influences transcription of *gna3 *[112] (Figure 2). Also GNA1 enhances cellulase gene expression, but the functions of these two G-alpha subunits are clearly different and suggest their response to distinct signals (C Seibel, G Gremel, RdN Silva, A Schuster, CP Kubicek, M Schmoll, Light dependent functions of the G-alpha subunit GNA1 of *Hypocrea jecorina (Trichoderma reesei*), *submitted*). Most importantly, cellulase gene expression in both G-protein mutant strains was still dependent on the presence of an inducer, thus ruling out binding of the inducer to a GPCR. Thus, these results indicate that the observed enhancing effect of cAMP [110] must be indirect.

## Conclusion

Our knowledge of how cellulase formation by *H. jecorina *is regulated has considerably advanced throughout the last 10 years, and the recently released genome sequence of *H. jecorina *[9] will further improve our understanding of why this fungus is superior to other organisms in its enzyme production. In addition, the current understanding of the process, as outlined in this review, will form a useful framework for genomic and transcriptomic analyses of various cellulase over-producing mutants, as currently performed in several laboratories worldwide. The next step will be the discovery of the regulatory processes altered during mutant isolation. It is also likely that such studies will identify additional cellular levels, bottlenecks and regulatory loops for cellulase formation in *H. jecorina *which have not yet been dealt with.

## Abbreviations

CAZy: classification system of carbohydrate active enzymes; CCR: carbon catabolite repression; GPCR: G-protein coupled receptor; QM: Quartermaster.

## Competing interests

The authors declare that they have no competing interests.

## Authors' contributions

CPK drafted the paragraphs on induction by cellulose and CCR and wrote the final version of the manuscript. MM drafted the paragraphs on transcriptional regulation. BS drafted the paragraphs on induction and regulation by lactose. MS and AS drafted the paragraphs on signalling. All the authors approved the final version of the manuscript.
